# Exploring Senior High School Students’ English Learning Demotivation in Mainland China

**DOI:** 10.3389/fpsyg.2022.822276

**Published:** 2022-02-15

**Authors:** Lixiang Gao, Honggang Liu, Xiaoxi Liu

**Affiliations:** ^1^Faculty of Education, Northeast Normal University, Changchun, China; ^2^Bozhou No. 18 Senior High School, Bozhou, China; ^3^School of Foreign Languages, Northeast Normal University, Changchun, China; ^4^School of Foreign Languages, Beijing Forestry University, Beijing, China

**Keywords:** English learning demotivation, teacher knowledge, important others, teacher responsibility, learner-related factor, learning contents, critical incidents

## Abstract

In the last 20 years, much attention has been paid to learners’ demotivation. Researchers have conducted many studies on second/foreign language learning demotivation from the perspectives of social culture, social psychology, and so forth. In China, related studies have mainly focused on college students’ demotivation; scant attention has been paid to senior high school students. Regarding scale development, although much progress has been made, there remains a need for a scale with high reliability and validity that is suitable for students in the basic education stage. Therefore, based on previous studies and choosing Chinese senior high school students as participants, this research study developed a scale with 55 items, and exploratory factor analysis (EFA) was used to develop a 28-item scale with six dimensions. The six-dimensional construct encompasses teacher knowledge, important others, teacher responsibility, learner-related factors, learning contents, and critical incidents, which are the key factors leading to English learners’ demotivation. Among them, the factor of critical incidents is new and has been overlooked by other researchers. Moreover, the descriptive analysis demonstrated the degree to which the demotivators influence learners, and the independent samples *t*-test found a significant difference in the impact of critical incidents in terms of the students’ language proficiency. Ultimately, four suggestions are put forward to remotivate and sustain learners’ motivation.

## Introduction

There has been increasing interest in demotivation over the past 20 years. After Dörnyei (2001) investigated the definition of second language (L2) learning demotivation for the first time, more and more researchers have paid close attention to L2 learning demotivation (e.g., Falout and Maruyama, 2004; Kikuchi, 2009; Karaca and Inan, 2020). From the perspective of the cognitive approach and social psychology, researchers have expanded on the factors that negatively influence L2 learners’ motivation, that is, the demotivators (Falout et al., 2009; Kim, 2011; Xaypanya et al., 2017). In China, with the promotion of new curriculum reform and the publication of the National English Curriculum Standards for General High School (2017), higher requirements for students’ English learning have been proposed. Consequently, senior high school students are expected to realize the significance of learning English fully and correctly and have “intense learning motivation” (Ministry of Education of the People’s Republic of China, 2018, p. 120). Nevertheless, most senior high school students currently suffer from English learning demotivation (Sakai and Kikuchi, 2009; Gao and Liu, 2015; Karaca and Inan, 2020). While some studies have addressed the existence of students’ English learning demotivation (Falout and Maruyama, 2004; Karaca and Inan, 2020), they mainly focused on college and elementary students’ demotivators and few have conducted an in-depth exploration of Chinese senior high school students’ demotivation. Therefore, choosing Chinese senior high school students as participants, the present study aimed to develop an adapted scale and explore the construct of their demotivation.

## Literature Review

### Defining Demotivation

It has been nearly 30 years since Chambers (1993) first introduced demotivation research into foreign language education. A few scholars have attempted to define demotivation (e.g., Dörnyei, 2001) and to identify the demotivators, in some empirical research studies. Dörnyei (2001) believed that demotivation “concerns specific external forces that reduce or diminish the motivational basis of a behavioral intention or an ongoing action” (p. 143). It is evident that this definition focuses on the external factors that lead to learners’ demotivation, which means learners used to be intensely motivated but gradually lost interest due to external forces. Nine demotivators were summarized in a qualitative study (Dörnyei, 1998), that is, the teacher (personality, commitment, competence, and teaching method), inadequate school facilities (the group is too big or not the right level; frequent change of teachers), reduced self-confidence (experience of failure or lack of success), negative attitude toward the L2, compulsory nature of L2 study, interference of another foreign language being studied, negative attitude toward the L2 community, attitudes of the group members, and the coursebook. Dörnyei and Ushioda (2021) developed this definition and thought of demotivation as “a negative process that reduces or diminishes a person’s motivation in relation to a behavioral intention or an ongoing action” (p. 140). This updated definition no longer emphasizes the external demotivators, but it treats demotivation as a negative process in which external and internal demotivators work together to make learners demotivated. We think the updated definition conforms to the mindset held by most researchers, which is that demotivation is a complex and negative process where external and internal factors correlate and interact with each other, thus causing learners’ demotivation.

### Demotivators in Second Language Learning

Many previous Social Science Citation Index papers and books addressing L2 learning demotivation in English were retrieved through our careful selection. In addition, some key and well-acknowledged Chinese papers were also referred to.

After a thorough analysis of the prior research that was retrieved from our literature search, it was found that empirical research on L2 learning demotivation has gradually increased (Tanaka, 2017; Zeynali et al., 2019; Li, 2021). While participants from different learning phases were involved in these studies, most of them were college students (e.g., Huang, 2012; Hassaskhah et al., 2015; Wang and Guan, 2020) and elementary school pupils (e.g., Kim, 2011; Kim and Seo, 2012; Kim et al., 2018). Previous research mainly focused on exploring demotivators (see Table 1), which were thought to consist of internal and external factors. The former consisted of learner-related factors, while the latter included teacher-related, environment-related, and course-related factors.

**TABLE 1:** Details of some articles exploring second language (L2) learning demotivation.

Author(s)	Sample and setting	Measures	Findings
Falout and Maruyama, 2004	164 Japanese freshmen	49-item questionnaire	(1) Teachers (2) Courses (3) Attitude toward L2 community (4) Attitude toward L2 itself (5) Self-confidence (6) Attitude of group members
Falout et al., 2009	900 Japanese college students	52-item questionnaire	(1) External conditions (2) Internal conditions (3) Reactive behaviors
Sakai and Kikuchi, 2009	656 Japanese students attending four Japanese senior high schools	35-item demotivation questionnaire	(1) Learning contents and materials (2) Teachers’ competence and teaching styles (3) Inadequate school facilities (4) Lack of intrinsic motivation (5) Test scores
Hassaskhah et al., 2015	308 Iranian English major undergraduate students (82 males and 226 females)	77-item demotivation test battery (DeMTB) questionnaire	(1) Institution related (2) Significant others related (3) Self-related
Xaypanya et al., 2017	158, 2–4 years undergraduate students in the Lao PDR	Self-developed questionnaire	(1) Difficulty to achieve linguistic accuracy (2) Negative attitudes toward English (3) Curriculum issues (4) Lack of supports and resources (5) Foreign language anxiety
Husniyah, 2019	190 Madrasa students in the Indonesian EFL context	A 25-close-ended questionnaire One open-ended question	(1) The nature of the target language (2) Lesson-specific factors (3) Learning materials (4) Teacher-related factors (5) The learning environment

Learner-related factors refer to the fact that learners’ motivation will diminish if they suffer from reduced confidence and low interest (Dörnyei, 2001; Sakai and Kikuchi, 2009; Liu and Ying, 2013; Albalawi, 2017). Three aspects are demonstrated under this dimension. Reduced confidence is the first learner-related factor. Dörnyei (2001) believed that learners’ reduced confidence is not identical to low self-confidence; rather, it refers to a decrease in confidence from a high level to a low level. Learners are more likely to doubt themselves if they encounter learning setbacks that they cannot handle or if they are blamed for poor academic performances. A negative attitude about language learning is the second learner-related factor. Sometimes it is challenging for learners to realize the significance of learning a foreign language, and they may consider it difficult and useless to devote time to learning a foreign language (Xaypanya et al., 2017). Study anxiety is the third learner-related factor. Anxiety is a subjective feeling triggered by the autonomic nervous system. Learners will feel nervous and depressed when they experience great anxiety (Spielberger et al., 1983). However, a manageable level of anxiety can help students courageously tackle learning difficulties and challenges and actively seek solutions to problems. If too much anxiety haunts learners, they will care more about others’ opinions and tend to be shy, worried, and afraid of making mistakes when using English. Thus, they will avoid occasions in which they can use English (Horwitz et al., 1986).

According to Sakai and Kikuchi (2009), teacher-related factors consist of the teachers’ attitude, teaching competence, language proficiency, personality, and teaching style. In their study that included 656 Japanese senior high school students, Sakai and Kikuchi (2009) employed a 35-item questionnaire to investigate the students’ English learning demotivators. The result indicated that teaching competence and teaching style had a significant effect on the students’ motivation. Teaching competence involves the ability to master and apply thinking skills, to master and apply new learning and teaching methods, to manage the class and communicate with students, to master communication and information technologies and integrate them into teaching, to conduct research, and to evaluate academic achievements (Aghaie, 2006). Teaching style refers to the way teachers solve problems, handle tasks, and make decisions in the teaching process. It varies from person to person and sometimes from group to group (Sternberg, 1997). Berger (1974) noted that teaching style encompasses teacher-centered, student-centered, and discipline-centered, one of which exerts an influence on the students’ learning.

Environment-related factors include learning environment-related factors and social environment-related factors. Learning environment-related factors mainly include the hardware facilities (Dörnyei, 1998; Sakai and Kikuchi, 2009; Dörnyei and Ushioda, 2011) and the class teaching environment (Unal and Yanpar Yelken, 2016; Karaca and Inan, 2020; Wu et al., 2020). Lack of hardware facilities will restrict teachers from adopting advanced teaching methods (Ushioda, 1998). Teachers fail to take advantage of multimodal resources and are unable to present content from different angles. For example, Sakai and Kikuchi (2009) found that, in schools where there was a shortage of computers and no access to the internet, teachers could not obtain the latest network resources and were unable to present video or audio resources to students in class. The classroom environment, especially the teaching environment, also has an impact on students’ demotivation, such as a delayed check of the assigned tasks (Unal and Yanpar Yelken, 2016), ignoring the students’ leading roles in the learning process (Berger, 1974), and failing to construct an atmosphere in which students can actively engage in learning English (Karaca and Inan, 2020).

Social environment-related factors include economic support, intellectual support, and the influence of other people. Parental economic investment, parent-child communication, and parents’ tutoring and supervision have been found to have a significant influence on students’ academic performance (Sui-Chu and Willms, 1996; Liu et al., 2020), leading to changes in their learning motivation. For example, in a study with 6,301 Korean pupils, Kim (2011) used a questionnaire to explore how after-class English tutoring affected the students’ motivation. The results showed that the after-class English tutoring based on financial support would enhance students’ learning motivation, expectations, and satisfaction. Moreover, students’ English learning motivation has been found to be influenced by the peers and family members that are most intimately associated with them (Hassaskhah et al., 2015).

Curriculum-related factors mainly consist of the characteristics of the English language, the course setting, and the learning contents (Falout and Falout, 2005; Li, 2021). First, as a curriculum-related factor, English is a subject that requires a significant amount of recitation and memorization. However, many students are reluctant to memorize and recite their English knowledge, and they gradually lose interest in learning English. For example, in a study with 52 Japanese college students, Kikuchi (2009) explored the participants’ demotivators in learning English when they were in high school. The results showed that the learners would suffer from demotivation if they were asked to memorize too many language points, such as too many new words, idioms, and passages. As curriculum-related factors, course setting and teaching contents refer to the arrangement of English lessons and the choice of teaching materials. For instance, Husniyah (2019) conducted a study of 190 Indonesia Madrasa students to investigate their English as a foreign language (EFL) demotivational factors. The result indicated that the nature of the target language and learning materials had a significant negative effect on the students’ motivation. Uninteresting learning topics and out-of-date learning materials did not satisfy the students’ needs and diminished their interest in learning.

In conclusion, regardless of whether previous researchers used a quantitative or qualitative methodology, they achieved consensus on learners’ L2 learning demotivation, so that two broad categories of demotivators emerged: external and internal demotivators (Falout et al., 2009; Kim, 2011). The external demotivators consisted of three aspects: the teacher, the environment, and the course contents. The internal demotivator only included one aspect, which was related to the learners themselves. However, some research gaps still exist that need to be filled. First, the instruments need to be improved. Researchers often refer to existing questionnaires or develop new ones as a tool to investigate learners’ demotivators, but few have confirmed the reliability and validity of those tools. Therefore, the results lack reliability (Liu and Ying, 2013). Second, empirical research on senior high school students’ demotivation should be enriched. The majority of studies investigated college or elementary students’ English learning demotivation (e.g., Falout and Maruyama, 2004; Kim, 2011; Chu, 2014; Kim et al., 2018; Karaca and Inan, 2020). For example, Falout and Maruyama (2004) investigated 164 Japanese college freshmen’s EFL demotivation and six demotivators emerged as teachers, courses, attitude toward L2 community, attitude toward L2 itself, self-confidence, and attitude of group members. Kim et al. (2018) investigated eight elementary EFL students’ English learning demotivation, and found teachers’ lack of clear delivery was the most prominent demotivator. However, there is a lack of in-depth attention to senior high school students’ EFL demotivation. Senior high school is an important transitional period from basic English learning in junior high school to advanced learning in college. They are confronted more challenges, such as the fierce competition in College Entrance Examination, the pressure from the high-loaded learning tasks, the more complicated knowledge to acquire, and critical psychological changes (Liu, 2010). All these visible and invisible factors may become the demotivators for senior high school students’ English learning. Though some previous studies has done the trial in unpacking the demotivation in senior high school English learning (e.g., Gao and Liu, 2015), there is still a large scope to explore in this field in the new era. This exploration is helpful to enrich the domain of language learning demotivation theoretically by finding out new potential demotivators. It’s also useful to offer the pedagogical implications for remotivating the demotivated learners. Lastly, few studies have investigated the critical incidents that occur during the students’ educational development. As individuals with their own ideas, students are continually developing and they tend to be affected by important incidents. However, few researchers have investigated the effect that critical incidents have on students’ ability to learn a foreign language. Consequently, the present study’s focus on Chinese senior high school students increases the understanding of how critical incidents influence students’ demotivation. Therefore, the present study aims to address three research questions:

(1) What are the demotivators in English learning for Chinese senior high school students?

(2) What are the levels of students’ English learning demotivators?

(3) Are there any significant differences in the demotivators in terms of gender and language proficiency?

## Materials and Methods

### Participants

The present study used an online questionnaire to collect data from Chinese senior high school students from three different grades. Of these participants (see Table 2), 462 were identified as being demotivated based on their answers to a self-assessment question. One hundred and fifty-nine (34.42%) were male and 303 (65.58%) were female. Moreover, 147 (31.82%) of them were from grade 1, 181 (39.18%) were from grade 2, and 134 (29.00%) were from grade 3. Following the traditional treatment of dividing students into HP and LP groups in studying students’ demotivation in terms of their language levels (e.g., Falout and Maruyama, 2004; Zoghi and Far, 2014; Kim et al., 2017), we used their entrance test scores for the new semester as the standard to do the group divisions. These participants were divided into two language proficiency groups: 301 low-proficiency (LP) students (65.15%) and 161 high-proficiency (HP) students (34.85%).^1^

**TABLE 2:** The information of participants.

	Gender		Grade			Proficiency	
	Male	Female	1	2	3	Low	High
Number	159	303	147	181	134	301	161
Percentage	34.42%	65.58%	31.82%	39.18%	29.00%	65.15%	34.85%

### Instrument

We built the item pool of the questionnaire in our study by referring to the previous survey studies (e.g., Gorham and Christophel, 1992; Chambers, 1993; Falout et al., 2009; Liu and Ying, 2013; Kikuchi, 2015) and transforming the interview data. The first author interviewed five students in Grade 2 in a high school face to face. Each interview lasted about 20 min and covered students’ English learning attitudes, the change of their motivation and the reasons for this change. The interviews were digitally recorded and transcribed. We went between the interview transcriptions and the items chosen from prior studies and added some new pieces which came from the interview data, for example, item 28 (My English teacher often found fault with me), item 53 (I did not want to learn English after our English teacher was changed) and so on.

The questionnaire, the English Learning Demotivators Scale (ELDS), used for this study is composed of three parts. The first part asks questions about the participants’ background, including grade, gender, and class level. The second part asks questions to identify whether the participants had experienced English learning demotivation. The last part was designed based on findings from previous studies (Gorham and Christophel, 1992; Chambers, 1993; Dörnyei, 2001; Falout et al., 2009; Kikuchi, 2015; Karaca and Inan, 2020; Dörnyei and Ushioda, 2021). The final section has 55 statements, with responses based on the 6-point Likert scale, with 1–6, respectively, representing “totally disagree, strongly disagree, disagree, agree, strongly agree, and totally agree.”

### Data Collection and Analysis

The online questionnaire was administrated in 2021, during the late summer holiday and during the first 2 weeks of the Fall semester, lasting about 1 month. A total of 462 questionnaires were identified as valid, which met our set standard. We then conducted exploratory factor analysis (EFA) with all the selected data since “a basic rule of thumb is to have at least five times as many cases as variables entered into the factor analysis” (Ho, 2006, p. 207). Descriptive statistics and an independent samples *t*-test were then conducted. The detailed process and results are included in the “Results and Discussion” section.

## Results and Discussion

### Exploratory Factor Analysis of English Learning Demotivators Structure

To explore the dimensionality of the English learning demotivators and the factor structure of the constructs included in the ELDS, and to reduce the number of items, EFA was performed using SPSS 26.0 software. The Kaiser-Meyer-Olkin (KMO) (KMO = 0.880 > 0.70) and Bartlett’s test (*p* = 0.000) results were desirable. We then adopted the extraction method of Principal Axis Analysis and the rotation method of Direct Oblimin. The threshold of factor loading was set as 0.30; that is, a factor loading value lower than 0.30 would be suppressed. Following the suggestions of Ho (2006) and Hair et al. (2010), we deleted:

(1) Items with a factor loading lower than 0.30.

(2) Items with cross-loadings.

(3) Items that were theoretically or logically inconsistent with other items under the same dimensions.

During the analysis process, 27 items (items 7, 8, 12–21, 29–32, 36–40, and 46–51) were discarded, which resulted in a 28-item ELDS (see Table 3). Six factors were identified, accounting for 61.599% of the variance. The commonality of each item was greater than 0.37. Two kinds of reliability were assessed: the internal consistency (Cronbach’s alpha) of the global scale as well as its six factors. The Cronbach’s alpha for the ELDS and six factors were, respectively, 0.886, 0.880, 0.892, 0.879, 0.880, 0.883, and 0.878, suggesting high reliability.

**TABLE 3:** Exploratory Factor Analysis (EFA) results of the 28-item scale (Pattern Matrix).

Items	Factor 1	Factor 2	Factor 3	Factor 4	Factor 5	Factor 6	Commonalities
	Teacher knowledge	Important others	Teacher responsibility	Learner-related factor	Learning contents	Critical incidents	
Q02 My English teacher gave us boring instructions.	0.685						0.619
Q01 My English teacher hardly exposed us to foreign culture.	0.667						0.393
Q04 My English teacher spoke too much Chinese.	0.573						0.511
Q05 My English teacher hardly organized activities.	0.549						0.376
Q03 My English teacher assigned us too much homework.	0.538						0.451
Q06 My English teacher didn’t dismiss class on time.	0.482						0.470
Q26 My friends did not learn English.		0.806					0.650
Q24 My parents did not encourage me to learn English.		0.759					0.833
Q25 My parents considered it useless to learn English.		0.737					0.833
Q28 My English teacher often found fault with me.		0.698					0.564
Q23 My parents did not buy me electronic products to learn English.		0.662					0.550
Q27 My English teacher was hard to get along with.		0.576			−0.303		0.442
Q22 My parents did not buy me reference books.		0.552					0.520
Q10 My English teacher was responsible.			0.939				0.883
Q11 My English teacher was patient with us.			0.882				0.791
Q09 My English teacher was passionate.			0.781				0.623
Q42 I felt nervous when I took a test.				−0.877			0.768
Q43 I felt nervous when I failed to give an answer.				−0.779			0.700
Q41 I felt anxious when I couldn’t follow the passage.				−0.713			0.540
Q44 I felt less confident when learning English.				−0.695			0.635
Q45 I felt less confident when I got low scores.				−0.364			0.381
Q33 The English textbooks were at my level.					−0.677		0.752
Q34 There was too much vocabulary that l did not understand in the readings.					−0.675		0.805
Q35 Learning contents are far from reality.					−0.667		0.741
Q53 I did not want to learn English after our English teacher was changed.						−0.806	0.696
Q54 I did not want to learn English when my classmate dropped out of school.						−0.738	0.566
Q55 I did not want to learn English after choosing science or liberal arts.						−0.640	0.578
Q52 I did not want to learn English after entering high school.						−0.632	0.576
Cumulative %	24.898	42.809	50.495	55.115	58.606	61.599	— —
Reliability	0.880	0.892	0.879	0.880	0.883	0.878	0.886

Factor 1 consists of teaching contents (items 1, 2, and 3) and teaching strategies (items 4, 5, and 6), which can be subsumed under pedagogical content knowledge (PCK), the integration of subject knowledge and teaching knowledge (Shulman, 1987). As a key factor influencing learners’ demotivation (Falout et al., 2009), how well English teachers master their professional knowledge, teaching skills, and language proficiency will have a significant effect on learners’ attitudes and motivation (Kikuchi, 2009). Under factor 1, teaching contents and teaching strategies are on the basis of the teachers’ understanding of their professional knowledge (item 2), their students (items 3, 4), the coursebook (item 1), and various teaching methods (items 5, 6). According to Tsui (2003), this refers to teacher knowledge, which covers the instructors’ knowledge about the English as a subject, their students, the environment, the curriculum, and general pedagogy. Consequently, it is appropriate to label this factor “teacher knowledge”.

Factor 2 involves the learners’ parents, classmates, and teachers, who potentially have an inevitable impact on learners (items 22, 23, 24, 25, 26, 27, and 28). Learners are developing all the time, and it is necessary for them to interact with people around them (Bronfenbrenner, 1979). The learners’ mutual interaction with others will influence their autonomy, but sometimes they cannot make their own choices. Parents, classmates, and teachers are closely connected to learners. It is apparent that their attitudes and learning methods will affect learners to some degree (Deci et al., 1991; Sui-Chu and Willms, 1996; Liu, 2014). Parents’ negative attitudes about English (items 24, 25) and inadequate economic support (items 22, 23), classmates’ passive learning behaviors (items 26), and a disharmonious teacher-student relationship (items 27, 28) will contribute to learners’ demotivation. This is in line with Hassaskhah et al.’s (2015) finding that significant others include the learner’s teacher, classmates, friends, and family. Accordingly, factor 2 is labeled “important others”.

Factor 3 is related to teachers’ attitudes toward both English teaching and their students. This attitude is derived from their responsibility, which is considered to be a relatively stable personality characteristic (Lauermann and Karabenick, 2011). English teachers are supposed to be responsible for English teaching and for motivating their students, such as being enthusiastic about teaching (item 9), being patient with students (item 11), and being responsible in the students’ eyes. This is consistent with Lenk’s (2007) connotation of responsibility. Thus, factor 3 is labeled “teacher responsibility”.

Factor 4 is about the learners’ psychological state, including their study anxiety (items 41, 42, and 43) and confidence in learning English (items 44, 45). When suffering from anxiety, learners are more likely to forget what they have mastered and sometimes they even make mistakes, thus influencing their English learning (Horwitz et al., 1986; Dörnyei and Ryan, 2015). Self-confidence, which highlights learners’ belief in their ability, is also an important demotivator. When learners fail their exams and have disappointing academic performances, they tend to doubt their abilities and no longer believe in themselves. Consequently, their confidence is most likely decreased and they become demotivated to learn. As previously mentioned, both anxiety and reduced confidence embody changes in an individual’s psychological wellbeing and will have an impact on learners’ demotivation. For example, to avoid embarrassment, a learner may feel too nervous to communicate with others in English after class (Kikuchi, 2015; Xaypanya et al., 2017); a learner with low confidence will have self-doubt and be demotivated to learn English (Falout et al., 2009; Hassaskhah et al., 2015; Karaca and Inan, 2020; Liu et al., 2020). These two aspects are specifically concerned with learners; thus, factor 4 is labeled “learner-related factor”.

Factor 5 indicates the influence that the learning contents have on learners’ motivation, including the influence of the characteristics of the coursebooks (items 33, 34) and the appropriateness of the learning contents (item 35). English teaching is not just about teaching the English language; it is also about the information conveyed through it. Therefore, the information should be appropriate to the level of the learners’ intelligence and satisfy their actual needs (Richards and Rodgers, 2001). When students encounter some difficulties, such as incomprehensible sentences and long passages, they have a tendency to have low motivation, that is, demotivation (Dörnyei, 1998; Sakai and Kikuchi, 2009; Gao and Liu, 2015; Husniyah, 2019). Because the characteristics of the coursebooks and the appropriateness of learning contents impact the learners’ motivation, factor 5 is labeled “learning contents”.

Factor 6 involves various events that occur during the learners’ different developmental stages and have a significant impact on their motivation to learn. These incidents are problems or challenges occurring in a certain context and exceed students’ capability, thus called critical incidents (Woods, 1993; NEPS, 2016; Joshi, 2018). It is likely that critical incidents are turning points in their life course and featured with unplanned, unanticipated, and influential (Richards and Rodgers, 2001). But not every incident is important to learners or influences their learning motivation. Only incidents that overwhelm their normal coping mechanisms play a key role in their development (Woods, 1993; NEPS, 2016), such as entering the next higher school (item 53), choosing science or liberal arts (item 52), classmates’ dropping out of school (item 55), and changing their English teacher (item 54). These significant incidents probably shape the learners’ psychological wellbeing and their English learning motivation. Tripp (2011) found that critical incidents have a significant impact on an individual’s development. Therefore, factor 6, which was not mentioned in previous studies, is labeled “critical incidents”.

**FIGURE 1:**
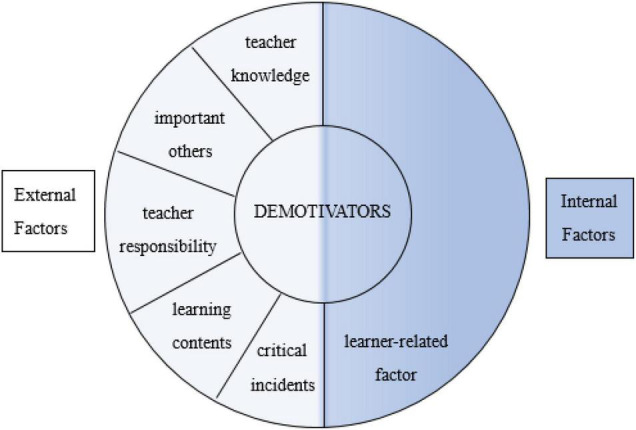
Classification of the demotivators.

These six factors can be classified further. It is evident that individual development is the outcome of the interaction between the external and internal factors (Feldman, 2006). Learners’ motivation is no exception. Falout et al. (2009) divided demotivators into the external conditions of learning and the internal conditions of the learner. The former included teacher immediacy, grammar-translation, and course level; the latter included self-denigration, value, and self-confidence. Kikuchi (2015) classified demotivators into internal and external forces. Internal forces consist of the learners’ loss of interest and experience of failure; external forces consist of the teachers, the characteristics of the classes, the class environment, and the class materials. Based on these findings, the present study supposes that senior high school students’ English learning demotivators fall into two categories: internal factors and external factors (see Figure 1). Specifically, the learner-related factor refers to the internal factors that impact learners’ motivation, and teacher knowledge, important others, teacher responsibility, learning contents, and critical incidents refer to the external factors. The internal and external factors work together to cause learners’ demotivation.

### Levels of Senior High School Students’ English Learning Demotivation

As is shown in Table 4, of the six demotivators, teacher responsibility is the most influential (*M* = 4.80; *SD* = 1.29), followed by the learner-related factor (*M* = 3.46; *SD* = 1.31), important others (*M* = 3.20; *SD* = 1.55), teacher knowledge (*M* = 3.04; *SD* = 1.11), critical incidents (*M* = 2.90; *SD* = 1.44), and learning contents (*M* = 2.45; *SD* = 1.56).

**TABLE 4:** The descriptive statistics of the levels of English learning demotivators.

Demotivators	*Min*	*Max*	*M*	*SD*
Teacher responsibility	1.00	6.00	4.80	1.29
Learner-related factor	1.00	6.00	3.46	1.31
Important others	1.00	6.00	3.20	1.55
Teacher knowledge	1.00	6.00	3.04	1.11
Critical incidents	1.00	6.00	2.90	1.44
Learning contents	1.00	6.00	2.45	1.56

*N = 462.*

Teacher responsibility was found to be the most impactful factor for most of the students. It really matters to students’ motivation whether teachers are responsible for or enthusiastic about teaching and whether they are patient with students. Teachers who are responsible will have a positive effect on students’ English learning motivation; teachers that are not responsible will have a negative effect on learning motivation. This is in line with Gorham and Christophel (1992), who found that English teachers’ attitude toward teaching and their students has a significant impact on motivating students. It is probable that teacher responsibility not only influences students’ motivation directly but causes a change in their psychological wellbeing.

Moreover, to some extent, the learner-related factor, important others, teacher knowledge, and learning contents all lead to students’ demotivation. This indicates that students’ English learning demotivation is the result of the interactions between the internal and external factors. First, the learner-related factor consists of study anxiety and reduced confidence (Albalawi, 2017). When learners encounter something that is beyond their ability, or they are in a situation where they feel uncomfortable (Horwitz et al., 1986), they are likely to feel nervous, anxious, and less confident. Furthermore, learners’ poor academic performance will discourage them from learning English (Zeynali et al., 2019), leading to demotivation. Second, important others are the people who are closely connected with learners, including their teachers, parents, and classmates. Parents’ negative attitude about English and inadequate economic support, classmates’ passive learning behaviors, and a disharmonious teacher-student relationship can result in learners’ demotivation. Third, teacher knowledge covers the instructors’ knowledge about how to teach and their subject knowledge (Sakai and Kikuchi, 2009). Specifically, the way teachers present knowledge and the teaching materials they select will draw the learners’ attention. If the teaching methods and materials are inappropriate, learners are more likely to be distracted. They tend to be bored and unwilling to learn English. Fourth, English teachers should prepare and present learning contents that are appropriate to the students’ proficiency level (Falout and Falout, 2005). Learners will be afraid of encountering difficulties and they will be demotivated when there are long passages and too many new words or when the learning topics are uninteresting and not relevant to real life (Kikuchi, 2009). In the present study, the learning contents factor was found to have a minor effect on students’ demotivation, which indicates that learners are capable of adjusting to the learning materials that are used.

Lastly, in the present study, critical incidents were found to exert a substantial effect on learners’ demotivation, which has been ignored in prior research. We know that there are significant differences in terms of the social and learning environments in which learners live and study. Some of the incidents that happen around them will shape their learning attitudes and behaviors (Woods, 1993). When their learning environment undergoes significant changes (such as entering the next higher school and choosing science or liberal arts), learners will need time and patience to adapt to the new and strange environment. If they fail to do so, they will be faced with negative emotions, and demotivation will occur. Student A mentioned the change of English teacher as the main demotivator in learning English.

I used to be fond of English and took an active part in the class. My English teacher was so humorous that I couldn’t wait to attend her class every day. However, she asked for maternity leave after teaching us for 2 months. A new teacher was assigned to replace her. He is very strict and nearly all students in my class, including me, fear him. All my English learning became messy, and I don’t know how to handle. I don’t like him and even get tired of his lessons. Now I hate to learn English. How I wish our former teacher could come back for us (Students A, 2021-9-8).

Student A was highly motivated in English learning as he was “fond of English” and “active in English class.” However, due to the occurrence of the unexpected critical incident, namely, the shift of English teacher, he became demotivated as he was “tired of his lessons” and “hate[d] to learn English.” This sudden event made him unprepared for what happened to him since he “didn’t know how to handle.”

### Comparison of Demotivators in Terms of Gender and Language Proficiency

The independent samples *t*-test was performed using SPSS 26.0 software to examine whether there were any significant differences in the impact of the six demotivators in terms of gender. The result indicated that there was no significant difference in terms of the learners’ gender (see Supplementary Appendix 1).

Demotivators have been reported to have significant effects on learners’ language proficiency (Kim et al., 2017). Consequently, another *t*-test was performed to evaluate the differences between two groups of students of different language proficiency levels for each of the six factors. It is found that both LP students and HP students suffer from English learning demotivation. Besides, under the influence of critical incidents, LP students are more likely to be demotivated than HP students (*t* = 0.629, *df* = 460, *p* < 0.05) (see Supplementary Appendix 2). Thus, it is probable that HP students will tackle learning difficulties bravely and seek solutions to these challenges (Falout and Maruyama, 2004). They tend to take advantage of these challenges and improve their performance.

## Conclusion and Implications

Using EFA, the present study explored L2 demotivation among Chinese EFL learners in senior high schools. It was found that most of the participants have experienced learning demotivation, and six demotivating factors emerged: teacher knowledge, important others, teacher responsibility, the learner-related factor, learning contents, and critical incidents. This study contributes to the current body of literature in that it identified a new demotivator, critical incidents, which was found to have a significant effect on learners’ demotivation. Previous studies usually treat learners as relatively stable and static individuals of a certain stage of development, and they ignore the effect that time has on them. The present study sheds light on the interactions between learners and critical incidents during different time periods. It views learners’ experiences through a four-dimensional perspective instead of a three-dimensional one by analyzing learners’ psychological changes and the factors influencing their English learning demotivation. Moreover, both LP students and HP students suffer from English learning demotivation, which shows that demotivation is a common phenomenon and it is experienced by students with various levels of language proficiency. Ultimately, the present study developed a questionnaire with high reliability and validity to investigate Chinese EFL learners’ English learning demotivation.

Based on the research findings, four proposals are put forward to intrigue, sustain, and enhance Chinese senior high school students’ motivation to learn English and decrease their learning demotivation.

First, as active and developing individuals (Bronfenbrenner, 1979), learners should seek a way to adjust their responses to the material being presented and the ways in which it is being taught, and they should build their confidence in their ability to learn English. It will be more beneficial if learners can accept, absorb, and value regulations (Khan et al., 2018). By means of introjected regulation, identified regulation, and integrated regulation, learners attempt to internalize their emotions and reactions, control their behavior, and be more autonomous, becoming more empowered to make choices and avoid internal conflicts. In this way, a certain type of study behavior can become attractive to them and maintained by them (Black and Deci, 2000). During this development process, learners will positively accept teachers, be engaged in class activities, and enhance their autonomy in class. Once they gain enough autonomy in the activities, they will experience a sense of achievement, build their confidence, relieve their feeling of anxiety, and become motivated to improve.

Second, English teachers should be more responsible for teaching and students. They are supposed to internalize assigned teaching goals, and increase their personal commitment, persistence, and higher quality of engagement (Ryan and Deci, 2000). As a result, teachers of strong responsibility tend to invest considerable efforts to prepare lessons of high quality and offer help to struggling students (Lauermann and Karabenick, 2011). Besides, they are likely to improve their teaching behaviors and enhance communication with students, thus facilitating teacher efficacy. In turn, teacher will stay positive and be fully engaged with English teaching and their students (Turner and Theilking, 2019). In addition, English teachers should be dedicated to improving their professional skills and become good examples for their students to follow. According to Shulman (1987), a good teacher should possess seven categories of knowledge, including PCK and knowledge of learners and their characteristics. English teachers are expected to combine English subject knowledge and teaching knowledge to become responsible and versatile teachers who have solid professional knowledge and a good command of using English; they should also adopt a variety of teaching methods. Furthermore, English teachers should establish a student-oriented class and be autonomy-supportive instructors. According to self-determined theory (SDT), an autonomy-supportive teacher who is in a position of authority will take the students’ perspective, acknowledge their feelings, and provide them with pertinent information and opportunities to make choices while minimizing the use of pressures and demands (Black and Deci, 2000). Thus, students will be more autonomous, and they will be able to speak for themselves. They will be encouraged to use the information to tackle a problem on their own. In turn, it is conducive to construct an environment in which student-centered learning is highlighted so that students can maintain or enhance their intrinsic motivation.

Third, English teachers should ensure their identities and integrate in-class and extracurricular activities to intrigue students. Language teachers own both professional and sociocultural identities, each of which will lead to their different efforts to choose what to teach (Gong et al., 2021). English teachers are expected not only to instruct students in English learning, but to expose students to excellent foreign cultures, which requires teachers to prepare both in-class and extracurricular activities. Specifically speaking, teachers can boldly organize some in-class activities for students, such as making a short speech, talent show, drama performance, etc., so that students can have the opportunity to use English and learn English independently. In return, teachers’ timely feedback on their performance is conducive to students’ adjustment and improvement of learning methods. In addition, teachers can organize colorful extracurricular activities, such as carrying out various forms of English competitions to stimulate students’ sense of self-esteem. Teachers can also rely on English clubs to expand classroom knowledge, enrich students’ school life, and improve their learning motivation.

Lastly, schools should guarantee access to modern technology and equipment so English teachers can integrate modern technology into their teaching design, thus making the lessons more interesting to students. With the development of educational informatization, technology is closely combined with teaching (Ghavifekr and Rosdy, 2015); this requires English teachers to utilize a teaching design that is not only based on the coursebook and students’ characteristics but also on technology. On the one hand, English teachers are capable of highlighting the instruction and contents of the learning tasks; on the other hand, they can make these tasks so appealing that students are willing to actively participate in them. Moreover, some necessary teaching infrastructures, such as flipped classrooms and smart classrooms, should be guaranteed to carry out advanced teaching modes (Wu et al., 2020). By implementing these teaching modes, teachers and students can interact more easily with each other and have a deeper understanding of teaching and learning with the help of big data.

This study has some limitations. It only had a small number of participants and included Chinese senior high school students; it failed to include students at different stages of language learning and distinguish demotivators among LP, intermediate, and HP students. It only focused on demotivation for English language learning; learning demotivation for other foreign languages was not investigated. The research instrument (the questionnaire) used in the study needs to be improved to guarantee its reliability and validity. Lastly, only EFA was performed; confirmatory factor analysis (CFA) was not done, thus limiting the impact of the data obtained from the questionnaire.

Further research is recommended to investigate a larger population of students at different stages of learning and in different countries, distinguish demotivators among LP, intermediate, and HP students, apply both quantitative and qualitative methods to obtain empirical evidence, and perform CFA to deeply understand the nature of foreign language learning demotivation in order to remotivate and sustain students’ learning motivation at a high level. Besides, we also suggest future research should be based on some psychological and educational psychology theories, such as SDT, to theoretically research demotivation.

## Data Availability Statement

The original contributions presented in the study are included in the article/Supplementary Material, further inquiries can be directed to the corresponding author.

## Ethics Statement

The studies involving human participants were reviewed and approved by the Faculty of Education, Northeast Normal University. Written informed consent to participate in this study was provided by the participants’ legal guardian/next of kin.

## Author Contributions

LG and HL: conceptualization, methodology, and writing. LG and XL: data analysis, review, and editing. All authors have read and agreed to the published version of the manuscript.

## Conflict of Interest

The authors declare that the research was conducted in the absence of any commercial or financial relationships that could be construed as a potential conflict of interest.

## Publisher’s Note

All claims expressed in this article are solely those of the authors and do not necessarily represent those of their affiliated organizations, or those of the publisher, the editors and the reviewers. Any product that may be evaluated in this article, or claim that may be made by its manufacturer, is not guaranteed or endorsed by the publisher.

## Footnotes

1 See the details in Section “Comparison of Demotivators in Terms of Gender and Language Proficiency.”
