# Supplementary material for: Exploring Senior High School Students’ English Learning Demotivation in Mainland China
Source: Front Psychol. 2022 Feb 15;13:822276. doi: 10.3389/fpsyg.2022.822276 (PMC8885720; doi:10.3389/fpsyg.2022.822276)
Supplement: Supplementary file 1 [file Data_Sheet_1.docx]

**Appendix 1**

The Independent Samples T-Test of English Learning Demotivators in Terms of Gender

| Factors | Variables | M | SD | p | t |
| --- | --- | --- | --- | --- | --- |
| teacher knowledge | male (159) | 3.23 | 1.07 | .290 | 2.691 |
|  | female (303) | 2.94 | 1.12 |  |  |
| teacher responsibility | male (159) | 4.81 | 1.18 | .164 | .125 |
|  | female (303) | 4.80 | 1.35 |  |  |
| important others | male (159) | 3.22 | 1.50 | .328 | .187 |
|  | female (303) | 3.19 | 1.57 |  |  |
| learner-related factor | male (159) | 3.63 | 1.34 | .782 | 2.028 |
|  | female (303) | 3.37 | 1.29 |  |  |
| learning contents | male (159) | 2.95 | 1.64 | .191 | 5.146 |
|  | female (303) | 2.19 | 1.45 |  |  |
| critical incidents | male (159) | 2.94 | 1.48 | .703 | .383 |
|  | female (303) | 2.89 | 1.42 |  |  |

**Appendix 2**

The Independent Samples T-Test of English Learning Demotivators in Terms of Language Proficiency

| Factors | Variables | M | SD | p | t |
| --- | --- | --- | --- | --- | --- |
| teacher knowledge | LP students (301) | 3.02 | 1.14 | .241 | -.386 |
|  | HP students (161) | 3.07 | 1.06 |  |  |
| teacher responsibility | LP students (301) | 4.76 | 1.33 | .203 | -.883 |
|  | HP students (161) | 4.88 | 1.23 |  |  |
| important others | LP students (301) | 3.21 | 1.52 | .243 | .200 |
|  | HP students (161) | 3.18 | 1.60 |  |  |
| learner-related factor | LP students (301) | 3.52 | 1.29 | .242 | 1.541 |
|  | HP students (161) | 3.33 | 1.35 |  |  |
| learning contents | LP students (301) | 2.42 | 1.55 | .974 | -.567 |
|  | HP students (161) | 2.51 | 1.56 |  |  |
| critical incidents | LP students (301) | 2.94 | 1.37 | .012 | .629 |
|  | HP students (161) | 2.84 | 1.57 |  |  |
